# SARS-CoV-2 Virus-like Particles Produced by a Single Recombinant Baculovirus Generate Anti-S Antibody and Protect against Variant Challenge

**DOI:** 10.3390/v14050914

**Published:** 2022-04-27

**Authors:** Edward Sullivan, Po-Yu Sung, Weining Wu, Neil Berry, Sarah Kempster, Deborah Ferguson, Neil Almond, Ian M. Jones, Polly Roy

**Affiliations:** 1 Department of Pathogen Molecular Biology, London School of Hygiene and Tropical Medicine, London WC1E 7HT, UK; edward.sullivan@lshtm.ac.uk (E.S.); po-yu.sung@lshtm.ac.uk (P.-Y.S.); weining.wu@lshtm.ac.uk (W.W.); 2 Division of Infectious Disease Diagnostics, National Institute for Biological Standards and Control, Potters Bar EN6 3QG, UK; neil.berry@nibsc.org (N.B.); sarah.kempster@nibsc.org (S.K.); debbie.ferguson@nibsc.org (D.F.); neil.almond@nibsc.org (N.A.); 3 School of Biological Sciences, University of Reading, Reading RG6 6AH, UK; i.m.jones@reading.ac.uk

**Keywords:** SARS, COVID-19, coronavirus, baculovirus, VLP, recombinant, antigenicity, immunogenicity, neutralizing antibody, protection

## Abstract

Coronavirus Disease 2019 (COVID-19), caused by infection with Severe Acute Respiratory Syndrome Coronavirus 2 (SARS-CoV-2), has highlighted the need for the rapid generation of efficient vaccines for emerging disease. Virus-like particles, VLPs, are an established vaccine technology that produces virus-like mimics, based on expression of the structural proteins of a target virus. SARS-CoV-2 is a coronavirus where the basis of VLP formation has been shown to be the co-expression of the spike, membrane and envelope structural proteins. Here we describe the generation of SARS-CoV-2 VLPs by the co-expression of the salient structural proteins in insect cells using the established baculovirus expression system. VLPs were heterologous ~100 nm diameter enveloped particles with a distinct fringe that reacted strongly with SARS-CoV-2 convalescent sera. In a Syrian hamster challenge model, non-adjuvanted VLPs induced neutralizing antibodies to the VLP-associated Wuhan S protein and reduced virus shedding and protected against disease associated weight loss following a virulent challenge with SARS-CoV-2 (B.1.1.7 variant). Immunized animals showed reduced lung pathology and lower challenge virus replication than the non-immunized controls. Our data suggest SARS-CoV-2 VLPs offer an efficient vaccine that mitigates against virus load and prevents severe disease.

## 1. Introduction

SARS-CoV-2 is the etiological agent of COVID-19. Since its initial identification in Wuhan, China, the virus has spread worldwide resulting in, to date, over 500 million confirmed cases and 6 million deaths. Genetic analysis of SARS-CoV-2 has shown that it shares 79.5% sequence homology with SARS, which emerged in 2003 [1].

SARS-CoV-2 is a member of the *Betacoronavirus* genus within the family *Coronaviridae*. The particle consists of a single copy of the 29.9 kb positive sense single stranded RNA genome associated with the viral nucleocapsid protein (N) which is surrounded by an envelope derived from the host cell plasma membrane, gained at the time of virus budding, embedded with 3 further viral structural proteins, the Spike (S), Membrane (M) and Envelope (E) proteins [2]. In addition to the structural proteins, coronaviruses express a large number of non-structural proteins, essential for replication but not present in the virus particle [3].

The most abundant of the Coronavirus (CoV) envelope-associated proteins is the M protein, a triple membrane-spanning glycoprotein that controls the conformation of the viral envelope [4]. The M protein oligomerizes to form lattices on the membranes of the ER-Golgi intermediary compartments, resulting in membrane distortion [3]. In addition, M directly interacts with the other coronavirus structural proteins enabling their recruitment into the nascent virus particle, such that the M protein is considered the primary driver of coronavirus assembly [2,5]. The envelope associated E protein is a small, 12 kDa protein, which some studies have shown to be a viroporin [5]. Very little E is incorporated into nascent virions, the majority remaining in the ER [6] but its absence impairs virus budding and leads to an attenuated phenotype [5,7,8]. Co-expression of E and M in isolation is sufficient to cause the formation and release of VLPs [5,9,10], which can also incorporate the S protein if it is co-expressed in the same cells [9,11,12]. The trimeric S protein is a type 1 transmembrane glycoprotein responsible for receptor binding and membrane fusion [13]. S binds to angiotensin conversion enzyme II (ACE2) on the host cell surface via the outermost S1 domain [14,15] leading to serine protease TMPRSS2 dependent cell entry driven by the S2 domain [16]. Both S and the nucleocapsid protein, N, are immunodominant antigens in SARS-CoV-2 infection and antibodies that block the interaction between S1 and ACE2 are neutralizing antibodies [17,18]. All currently approved vector-based or RNA vaccines for COVID-19 rely solely on S for their protective responses [19].

Previously, we described VLPs for SARS-CoV following expression of the requisite structural proteins in insect cells using a recombinant baculovirus [9]. The VLPs mimics authentic virus structure, including the trimeric S protein structure, and offer multiple presentation of the trimeric S protein to stimulate humoral immunity as well as uptake by antigen presenting cells for the generation of T-cell responses [20,21]. Several VLP based vaccines for other viruses, produced by the baculovirus insect cell system, are currently in use, proving evidence of scalability and acceptability [22,23].

Here we report the generation of SARS-CoV-2 VLPs based on the co-expression of S, M and E proteins in insect cells. Purified VLPs presented as heterogeneous vesicular structures which were strongly recognized by COVID-19 convalescent human sera. Small mammal immunization trials generated neutralizing antibodies that protected against disease induced by subsequent variant live virus challenge. Our data demonstrate that SARS-CoV-2 VLPs produced by an established expression technology offer a potential route to a vaccine for COVID-19.

## 2. Materials and Methods

### 2.1. Cell Lines

*Spodoptera frugiperda* (Sf9) cells were purchased from Thermo Fisher Scientific (cat. 11496015, Waltham, MA, USA). *Trichoplusia ni* (Tnao38) cells [24] were a gift from Gary Blissard, Boyce Thompson Institute at Cornell University, US. Both cells were maintained in Insect-XPRESSTM Medium containing L-glutamine (Lonza, cat. BELN12-730Q, Basel, Switzerland) and 2% FCS in shaking conical flasks at 28 °C.

### 2.2. Construction of Recombinant Baculoviruses

A sequence encoding the S protein of the original Wuhan SARS-CoV-2 isolate (QHD43416.1), codon optimized for *Spodoptera* cells, was obtained from Integrated DNA Technologies as described [25] and cloned in place of the resident SARS S protein in vector pACVC3-SARS-S-E [9], leaving the E open reading frame unchanged. Similarly, an Spodoptera codon optimized SARS-CoV-2 M gene was purchased from Eurofins Genomics and cloned into the baculovirus expression vector pAcYB2 [26]. A single baculovirus expressing the 3 proteins was generated by co-transfection of pACVC3-SARS-E-Covid19-S and pAcYB2-Covid19-M with *Bsu36I* linearized AcMNPV DNA [27].

### 2.3. Expression and Purification of VLPs

*Spodoptera frugiperda* (Sf9) cells were used for generation of recombinant baculoviruses and for subsequent virus amplification. For production of VLPs *Trichoplusia ni* (*Tnao38)* cells [28] were used. Virus was grown at an MOI of 0.01 and infected cells were incubated for up to 6 days before harvesting the supernatant. Virus infection for protein expression and VLP assembly employed an MOI of 5 and incubation for 4 h, after which the cells were pelleted and re-suspended in fresh culture medium to maximize expression levels [29,30]. Infected cells were then incubated for a further 72 h by which time cell viability measured by trypan blue exclusion was ~50%. The resultant supernatant was harvested, clarified by centrifugation twice at 4500× *g*, 4 °C for 20 min, and the VLPs then pelleted by centrifugation through a 25% sucrose cushion, at 100,000× *g*, 4 °C for 100 min. VLPs were re-suspended in 5 mL of PBS and purified by centrifugation on a 20–60% sucrose gradient at 100,000× *g*, 10 °C for 18 h. The gradients were fractionated and the presence of S protein assessed by dot blot with CR3022 (Absolute Antibody, Oxford, UK). The pixel density in each dot was assessed using Image J [31].

### 2.4. SDS-PAGE and Western Blot

Samples were resolved on Bolt^TM^ 4–12% Bis-Tris Plus gradient gels (Invitrogen, Waltham, MA, USA). Gels were either stained with Coomassie brilliant blue or subjected to Western blot using appropriate antibodies: rabbit polyclonal anti-SARS-CoV-2 S antibody (Abcam ab272504, Cambridge, UK) or rabbit polyclonal anti-SARS-CoV/CoV2 M antibody (Novus Biologicals NB100-56569, Littleton, CO, USA).

### 2.5. Transmission Electron Microscopy (TEM)

Peak fractions from the sucrose gradient, identified by western blot with an anti-S antibody, were diluted 4-fold in PBS and concentrated to 10% of their original volume using spin filters with a cut-off of 1MDa. Carbon coated formvar grids were floated on droplets of the concentrated samples for 5 min followed sequentially by 5 min on a droplet of water and then on 1% uranyl acetate. The grids were blotted and allowed to dry before examination in a JEOL 2100 Plus microscope operating at 200 kV.

### 2.6. ELISA Test

For the tests of VLP antigenicity, 96-well Nunc Maxisorb plates (Thermo Fisher Scientific) were coated with 0.5 μg of purified VLPs per well diluted in 50 μL of carbonate coating buffer (15 mM Na_2_CO_3_, 36 mM NaHCO_3_, pH 9.6) and incubated overnight at 4 °C. The plates were washed with PBS containing 0.05% Tween-20 (PBST) and blocked with Sea Block blocking buffer (Thermo Fisher Scientific) for 1 h at room temperature. 2-fold serial diluted human sera in blocking buffer were added to the plates and incubated for 1 h at room temperature. After three washes, the bound antibody was detected with goat anti-human Ig (γ chain specific)-HRP conjugated secondary antibody (Thermo Fisher Scientific) diluted 1:10,000 in blocking buffer. The assay reaction was developed with the 1-step Ultra TMB-ELISA substrate (Thermo Fisher Scientific). The reaction was left to develop for 5 min and then stopped with 2 M H_2_SO_4_. The optical density was determined at 440 nm using a plate reader. The neutralizing antibody response following immunization was measured using the “cPass™ SARS-CoV-2 Neutralization Antibody Detection Kit” (Genscript, L00847, Piscataway, NJ, USA). Used as directed, the assay detects functional neutralizing antibodies antibodies that prevent the receptor binding domain (RBD) of SARS-CoV-2 binding to ACE-2 expressed as % of blocking activity [18].

### 2.7. Immunogenicity Studies

Five female Golden hamsters, animals S92 to S96, were administered with 300 µL (10 µg total protein as determined by Bradford assay, containing ~2.5 µg S protein, determined by SDS-PAGE and Coomassie blue staining using a BSA standard) of non-adjuvanted VLP preparation under the skin on two occasions, 4 weeks apart. The fixed dose was based on previous adjuvant free small mammal immunization with insect cell expressed Influenza hemagglutinin [32] and is similar to that described for other candidate SARS-CoV-2 vaccines [33]. A control group of 5 hamsters, S97 to S101 received only buffer. Two weeks after the second immunization hamsters were challenged intranasally with 50 µL (1.5 × 10^5^ IU) of SARS-CoV-2 isolate B1.1.7, [34] equally distributed between the two nostrils. The B.1.1.7 virus stock, obtained from Public Health England at Porton Down, had been propagated to passage 3 on the Vero/hSLAM cell line and deep sequenced to confirm absence of attenuating mutations at the furin cleavage site. Following virus challenge, all animals were weighed twice daily (AM and PM). One hamster from each group was euthanized at 4 days post-challenge, two further animals from each group at 10 days post-challenge and the final two from each group at 14 days post-challenge. Oral swabs were taken from each animal at days 0, 1, 2, 3, 4, 7, 10 and 14 into Virus Transport Medium (VTM) (Hanks balanced salt solution with 2% heat-inactivated fetal calf serum, penicillin/streptomycin, 0.5 µg/mL amphotericin B) for RT-qPCR analysis and dry swabs were taken for Point of Care (LFD) testing. Nasal swabs were taken at days 4, 7, 10 and 14 post-challenge. All procedures were carried out in a UK laboratory in accordance with Home Office licensed procedures. Statistical analyses were performed using Graph Pad Prism v.9 and SigmaPlot v12.5. All in vivo studies were performed under the auspices of the Animal (Scientific Procedures) Act 1986. Prior to starting the work a license to perform studies had been obtained from the Secretary of State. As part of the application for this license the proposal was submitted to the local Animal Welfare and Ethics Review Board and their agreement secured. Immunization studies were performed in purpose built facilities that operate at bio-containment level 2. Prior to virus challenge the subjects were transferred to a facility that operates at bio-containment level 3 and were held there until the end of the study.

### 2.8. qRT-PCR

Total nucleic acid was extracted from a 200 µL sample using the MagnaPure24 (Roche, Basel, Switzerland) External Lysis Pathogen 200 protocol with elution into 50 µL. qRT-PCR was performed in triplicate, with 5 µL per reaction, with primers and probe targeting the envelope protein as described by Corman et al. [35]. Viral shedding data was expressed in International Units per mL (IU/mL) calibrated against the WHO RNA standard for SARS-CoV-2 RNA (NIBSC: 20/146).

### 2.9. Culture from Swabs

20 µL of the VTM sample was added to duplicate cultures of VeroTMPRSS2 cells either neat or diluted 1:2 in VTM. After 3 days the supernatant was removed, and the cells fixed for 30 min with 4% neutral buffered formalin followed by staining with 0.5% methyl violet. Where cell death was observed the presence of SARS-CoV-2 in cell culture supernatant was verified by qRT-PCR as described above. A sample was considered positive by culture if at least one of the four replicate wells showed destruction of the cell monolayer and was positive for SARS-CoV-2 by qRT-PCR.

### 2.10. Point of Care Testing

The BioSensor SARS-CoV-2 Ag Kit (Oxford Biosystems, Milton, UK) was used in accordance with manufacturer’s instructions to process swab samples from hamsters. Sample were added to lateral flow buffer and applied to the device and the output scored according to the WHO scoring system (https://extranet.who.int/pqweb/vitro-diagnostics/performance-evaluation; accessed on 25 April 2022). The LFDs were imaged and the relative pixel density in the test to control lanes was measured using Image J [31].

### 2.11. Pathology

Intact lungs were collected at post-mortem, fixed for 72 h at room temperature in 10% neutral buffered formalin (Sigma-Aldrich, St. Louis, MO, USA) and embedded in paraffin wax (VWR) in accordance with standard histological processes. The samples were examined visually for pathology related markers and scored for severity on a scale of 1 to 4. In addition, four-micron thick sections were mounted on poly-L-lysine coated slides (VWR), de-waxed with xylene (Thermo Fisher Scientific) and re-hydrated via graded ethanol:water solutions (Thermo Fisher Scientific). Hematoxylin and eosin sections were evaluated for pathological changes associated with disease and assigned a score for each variable by two veterinary pathologists, independently. The samples were scored blinded.

### 2.12. Immunohistochemistry (IHC)

Immunohistochemical staining was performed using the Leica Bond Polymer Refine staining system (Leica Microsystems DS9800, Wetzlar, Germany). Onboard de-waxing was performed in accordance with the standard Leica Bond protocol and staining undertaken using IHC Protocol F with the following adaptations: additional non-specific block prior to primary antibody incubation (10% normal horse serum (Biorad, Hercules, CA, USA), 1× Casein (Vector Labs) in PBS) and extended hematoxylin staining time (10 min). Antibodies were diluted to their optimal staining concentration in Bond primary antibody diluent (Leica, AR9352) as follows: SARS-CoV-2 Spike protein: 1:1000 (Leica, AR9961) 30 minutes, (Rabbit PAb 40150-T62-COV2-SIB, Sino Biologicals, Beijing, China), SARS-CoV-2 Nucleoprotein: 1:2000 (Leica, AR9961) 30 minutes, (Mouse Mab 40143-MM05-SIB, Stratech Scientific/SinoBiologicals). For both antibodies antigen unmasking was undertaken using Heat Induced Epitope Retrieval (HEIR) solution 1 (Leica AR9961) for 30 min at 100 °C.

## 3. Results

### 3.1. Baculovirus Expression of SARS-CoV2 S, M and E Proteins Results in VLP Formation in Insect Cells

**Figure 1:**
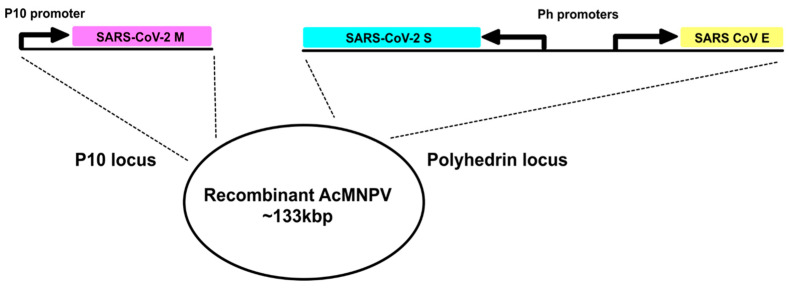
A schematic representation of the gene arrangements in the transfer vectors used to generate the recombinant baculovirus expressing SARS-CoV-2 VLPs. The SARS-CoV-2 M protein was expressed from the P10 promoter integrated at the P10 locus of the AcMNPV genome while S and E were expressed by back-to-back non-clashing polyhedrin promoters integrated at the polyhedrin locus of the genome.

**Figure 2:**
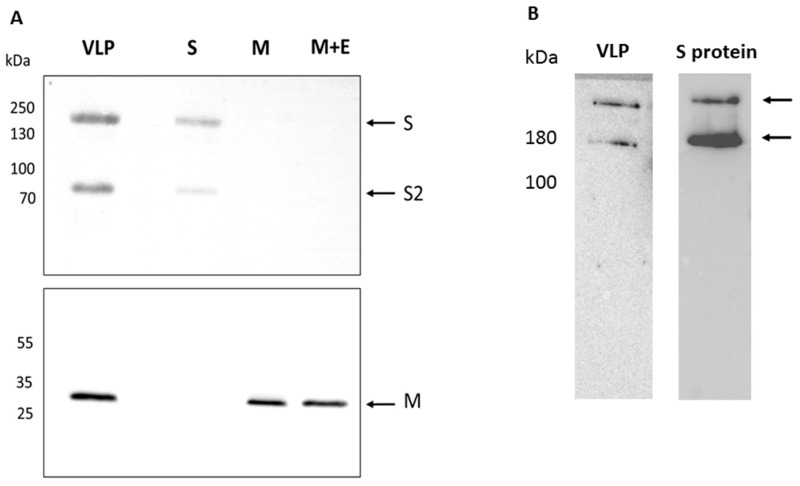
Expression of SARS-CoV-2 structural proteins by recombinant baculoviruses. (**A**) Individual recombinant virus infections of Sf9 cells were harvested at 2 days post infection and total cell extracts analyzed by SDS-PAGE and Western blot with appropriate antibodies. The lanes are: VLP—virus expressing structural proteins S, M and E; S—virus expressing S only; M—virus expressing M only; M + E—virus expressing M and E. The upper panel was probed with an anti-S antibody and the lower panel probed with an anti-M antibody. (**B**) Western blot of cell extracts from virus infected cells expressing S only or S as part of the VLP construct after electrophoresis under conditions of low denaturation. The position of bands with molecular masses consistent with monomer and trimer are indicated. Markers to the left of the gels are in kilodaltons.

A recombinant baculovirus driving the expression of SARS-CoV-2 S and M proteins from the polyhedrin promoter and the SARS-CoV E protein from the p10 promoter was constructed as described (Section 2 and Figure 1) and the presence of each coding region in the final recombinant virus confirmed by DNA sequencing. Individual recombinant viruses expressing S, M or M + E were also constructed. The SARS-CoV and SARS-CoV-2 E proteins share 97% homology and are functionally exchangeable for the purposes of VLP production. Moreover, only a low level of E is incorporated into virus particles and it is not required for the neutralizing antibody response [3,36]. Infection of *Spodoptera frugiperda* (Sf9) cells with each recombinant virus followed by immunoblotting at 3 days post infection confirmed the expression of the S and M CoV structural proteins separately and both proteins together in the case of the VLP competent construction. The recombinant viruses expressing S alone and the VLP expressed two bands identified with an S specific antiserum raised against a C-terminal peptide with molecular weights consistent with the complete S and the S2 cleavage product that were absent in cells infected with the M or M + E recombinant viruses (Figure 2A). S protein maturation in insect cells is consistent with furin cleavage as previously shown for the expression of the S protein alone using recombinant baculoviruses [37]. The proportion of maturation, as judged by band intensity, was~50% and was assumed not to impact immunogenicity, although this was not formally tested. Various forms of S, some of which retain the furin cleavage site and others that remove it, are used in the current range of available vaccines [38]. The M and M + E recombinant viruses expressed a band reactive with an M specific serum that was also produced by the recombinant baculovirus expressing VLPs. When cell extracts of recombinant virus infections expressing the VLP or single S protein were analyzed under conditions of minimal denaturation, both the monomer and trimer bands were apparent following Western blot (Figure 2B). Thus, the structural proteins required for SARS-CoV-2 VLP formation were produced by a single recombinant baculovirus as previously shown for SARS-CoV [9]. Formal demonstration of E expression was not possible due to the lack of an E specific serum.

**Figure 3:**
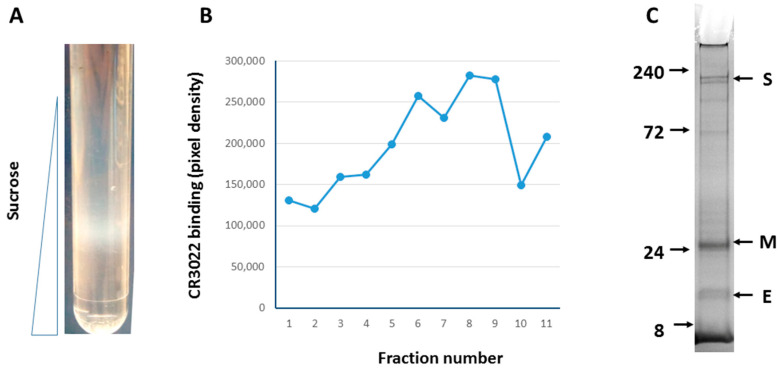
Purification of SARS-CoV-2 VLPs from infected *Tnao*38 cells. Infected cultures were processed as described and the resulting gradient (**A**) fractionated from the top. Each fraction was bot blotted to nitrocellulose, incubated with human anti-S monoclonal antibody CR3022 and developed with an anti-human HRP conjugate. The blot was scanned and the dot intensity recorded and plotted against fraction number (**B**). The peak fractions (#8 & #9) judged by appearance and immunoreactivity was analyzed by SDS-PAGE and stained with Coomassie brilliant blue R250, which showed the presence all three VLP proteins, S, M and E (**C**).

To generate and purify VLPs, large scale infection (10^9^ cells) of the more productive Tnao38 cells [24,28] was performed and the infected cell supernatant harvested at 72 h post infection when viability was ~50%. Cell debris was removed by low-speed centrifugation and VLPs remaining in the supernatant, ~100 μgs estimated by gel staining, were collected by ultracentrifugation. VLPs were further purified on a 20–60% sucrose gradient where they formed a distinct band of particulate structures at ~35% sucrose consistent with VLP presence (Figure 3A). The gradient was fractionated and probed for SARS-CoV-2 S antigen by SDS-PAGE and Western blot. The peak fraction that reacted most strongly with conformation dependent anti-S monoclonal antibody CR3022 (Figure 3B) showed 3 prominent bands at ~180, 30, and 10 kDa on Coomassie blue stained SDS-PAGE (Figure 3C). The observed molecular weights correspond to S, M, and E proteins respectively. Other proteins present at lower levels in the VLP preparation, none of which were major contaminants, were likely insect cell membrane proteins present at the sites of VLP budding and incorporated into budded VLPs, as previously described by others [39].

**Figure 4:**
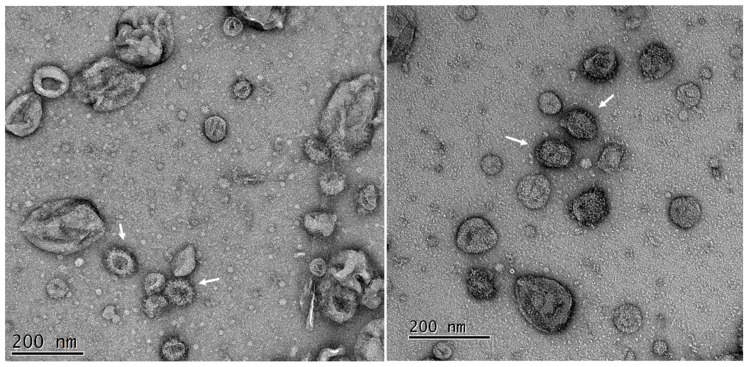
Transmission electron microscopy of purified SARS-CoV-2 VLPs purified from insect cells. Fractions were diluted to reduce the sucrose and re-concentrated before adsorption to carbon coated grids. The grids were strained with 2% uranyl acetate. Peak fractions (cf. Figure 3) of the VLPs showed multiple vesicle like structures among which are many with fringe like projections on their surface (arrowed). Two typical fields of the same adsorbed sample are shown.

Fractions from the ~35% sucrose fraction which reacted most strongly with the S monoclonal antibody were coated to carbon coated formvar TEM grids and analyzed by electron microscopy following staining with 1% uranyl acetate. Among the heterogeneous vesicles present were many vesicles of ~100 nm diameter that displayed a distinct fringe of ~10 nm in depth that were typical of the crown-like spikes which define coronavirus morphology [12,40]. The size and presentation of the visible spike was consistent with a trimeric S protein as imaged by others [41,42,43] supporting the biochemical data on expression and trimerization (cf. Figure 2B). Baculoviruses were absent from most fields of view consistent with their sedimentation to the bottom of the gradients used (Figure 4).

### 3.2. Antigenicity of SARS-CoV-2 VLPs

**Figure 5:**
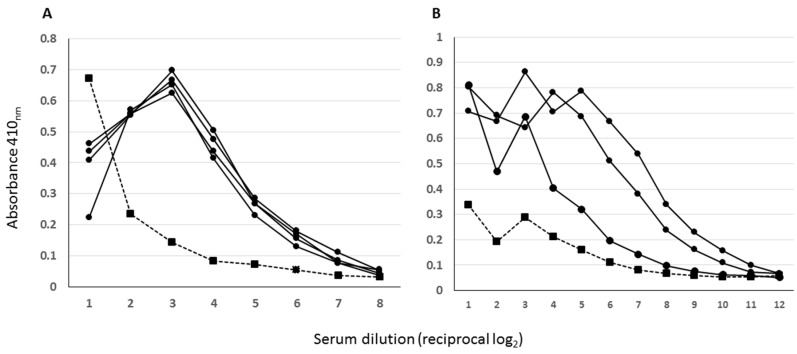
Antigenicity of SARS-CoV-2 VLPs determined by ELISA. Purified VLPs were adsorbed directly to the immunoplates overnight and blocked extensively before being probed with a twofold dilution series of pre-screened convalescent sera. Primary antibody binding was detected with an anti-human Ig HRP conjugate. (**A**)—Sera pre-screened by ELISA on purified SARS-CoV-2 S1 protein. (**B**)—Sera pre-screened by peptide array. In both assays filled circles and solid lines are sera that pre-screened positive while the single filled square dashed line is a negative. Some inhibition of binding at the high serum concentrations is apparent with the left panel serum set.

To assess relevant antigenicity, VLPs present in the peak fraction were coated to ELISA plates and probed with antibody positive convalescent sera previously screened using baculovirus expressed SARS-CoV-2 S1 protein [25]. Multiple convalescent sera but not a naïve serum control reacted strongly with immobilized VLPs (Figure 5A) although the serum set used showed some inhibition of binding at high serum concentrations, likely due to high concentrations of non-specific immunoglobulin. For the SARS-CoV-2 spike protein, extensive pepscanning of convalescent sera has shown that reactivity with linear epitopes is relatively poor [44,45] suggesting that VLPs used here display S in an antigenic form suitable for binding conformational antibody. To ensure that the sera used was not biased by pre-screening on insect cell expressed S1 protein, for example as a result of the high mannose glycan content of insect cell expressed proteins [46], VLP coated plates were also probed with a wholly different patient sera cohort pre-screened by peptide array [47]. As before, reactivity was high for sera pre-scored as positive for infection but not for the serum pre-scored as negative (Figure 5B). Thus, in keeping with their reactivity with conformational monoclonal antibody CR3022 and their appearance by TEM, insect cell derived SARS-CoV-2 VLPs are antigenic.

### 3.3. Immunogenicity of VLPs in Model Animals

**Figure 6:**
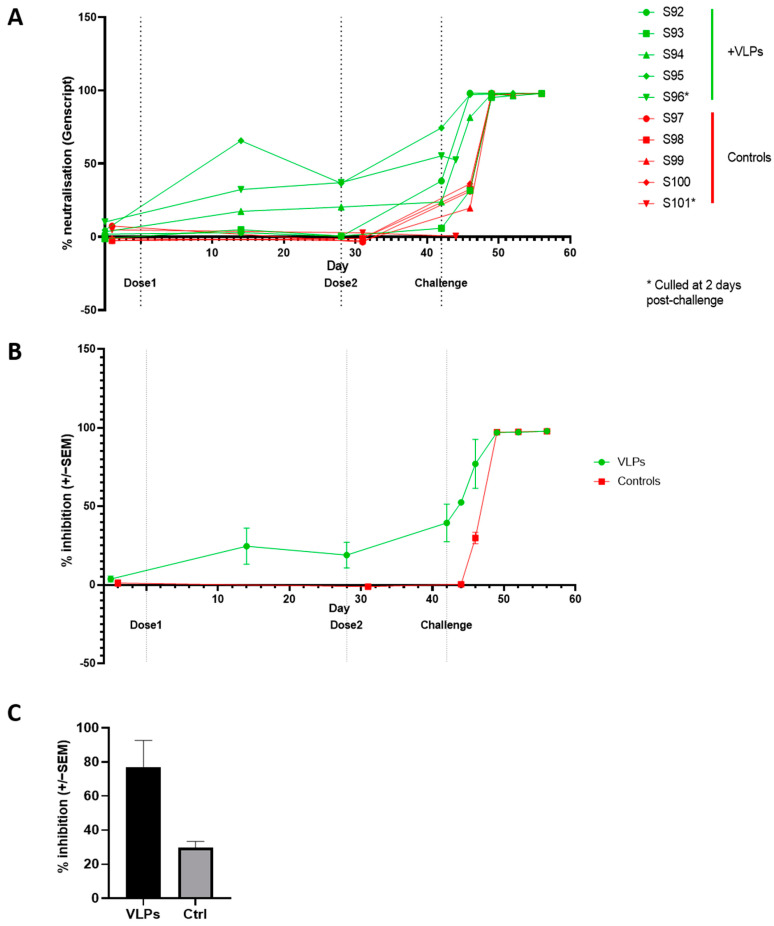
Neutralizing antibody responses to SARS-CoV-2 and vaccination. (**A**) Individual antibody titres throughout the experiment as determined by the cPass total neutralizing antibody ELISA and (**B**) mean of group +/− SEM. (**C**) At day 4 post challenge a single animal (S96) from the VLP treated group had a higher level of RBD blocking antibody than a control animal (S101). *—animals culled early.

As the composition, appearance and immune recognition of the VLP preparation derived from insect cells infected with the VLP recombinant baculovirus was consistent with previous studies of CoV VLPs [12,48,49], insect cell derived VLPs bearing the SARS-CoV-2 S protein were assessed as a candidate vaccine in a hamster challenge model [50,51]. Infection of Syrian hamsters with SARS-CoV-2 shows replication of the virus in the lungs with a pathology similar to that reported for human COVID-19 and a neutralizing antibody response that protects from disease [52,53]. Hamsters were vaccinated with gradient purified VLPs (n = 5, dose = 10 µg total protein per immunization containing ~2.5 µg S protein, see Material and Methods) and boosted with the same material at 4 weeks post prime. Five additional control animals received no treatment. Seroconversion and the development of neutralizing antibody were tested using an anti-RBD antibody competition immunoassay previously shown to perform as an accurate surrogate for measurement of functional neutralizing antibody responses [18] and was apparent in 4 out of 5 animals after the prime and in all animals in the immunized group after the boost (Figure 6A). No RBD competitive activity was found in the sera of the control group.

Two weeks after the second immunization, hamsters received a non-homologous intranasal challenge with a pre-titrated infectious dose of the SARS-CoV-2 B.1.1.7 variant and the infection was monitored by virus recovery and a virus antigen point of care lateral flow device using oral or nasopharyngeal washes from each animal. In addition, animals were weighed and scored for clinical signs as described [50]. Neutralizing antibody titers rose within 4 days of challenge in the VLP immunized group and later also in the control group (Figure 6B). A randomly chosen single animal (S96 from the immunized group and S101 from the control group) culled from each group at four days post challenge to provide an indication of the relative response of each group showed higher anti-RBD antibody titers in the VLP-immunized animal when compared with the control although no statistical significance could be attributed to the data (Figure 6C).

**Figure 7:**
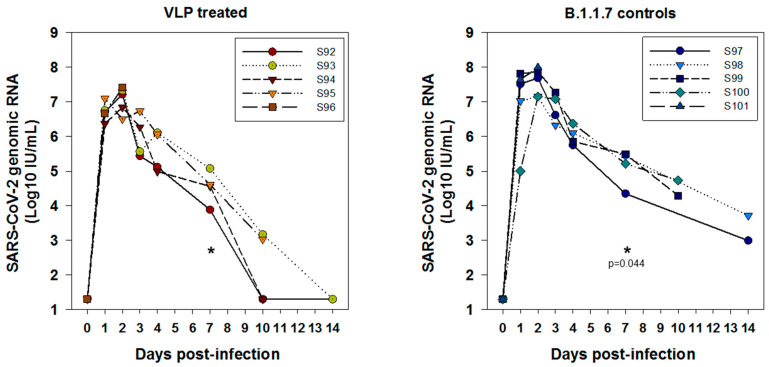
Viral genomic RNA kinetics in oral swabs demonstrating slower clearance of gRNA in oral swabs in controls compared to VLP treatment group. One-tailed t-test at day 4; *p* = 0.16 (ns); day 7, statistically significant *p* = 0.044 (indicated). Comparison of 2 day (peak) virus load in oral swabs was 1.4 × 10^7^ for the treatment peak compared to 4.7 × 10^7^ for the B.1.1.7 control (i.e., approximately half a log difference). It was non-significant by Mann-Whitney Rank Sum test (* *p* = 0.421).

The oral swabs of all animals from day 1 post challenge were positive for virus by genomic RNA RT-PCR but the kinetics demonstrated slower clearance of gRNA in oral swabs in controls compared to the VLP treatment group (Figure 7). A one-tailed t-test at day 4 was not significant (*p* = 0.16) but at day 7 there was a statistically significant difference between the groups (*p* = 0.044). The day 2 peak virus load in oral swabs was 1.4 × 10^7^ in the treatment group compared to 4.7 × 10^7^ in the control, about half a log lower but this was not significant by Mann-Whitney Rank Sum test (*p* = 0.421). A similar pattern was seen in nasal swabs: day 4: *p* = 0.35 (ns); day 7, *p* = 0.0232 (one tailed *t*-test) (not shown).

**Figure 8:**
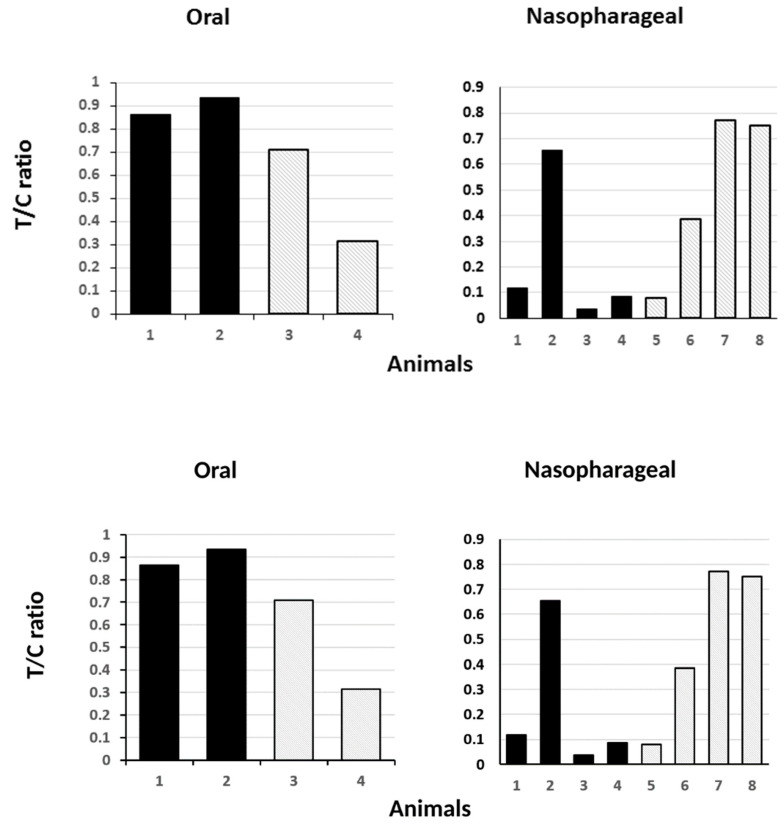
Presence of viral antigen in animals following live virus challenge. Oral and nasopharyngeal washes were subjected to point of care lateral flow device (LFD) detection of virus antigen and the test to control bar ratio was plotted. The data shown is for day two (**left panel**) and day four (**right panel**) post challenge. Only two animals from each group were tested by LFD at day 2. No virus antigen was detected by day 7 in either group. Solid filled bars—vaccinated group. Stipple bars—control group.

Virus antigen positivity by LFD of the oral swabs was also strongly positive for both immunized and control groups at day 2, reduced at day 4, with a trend towards lower antigen levels in the immunized animals (Figure 8) and absent by day 7 to the end of the experiment. Culturable virus was recovered from oral swabs throughout days 1 to 4 post-challenge with no discernible difference between the groups but not from any animal thereafter (not shown).

**Figure 9:**
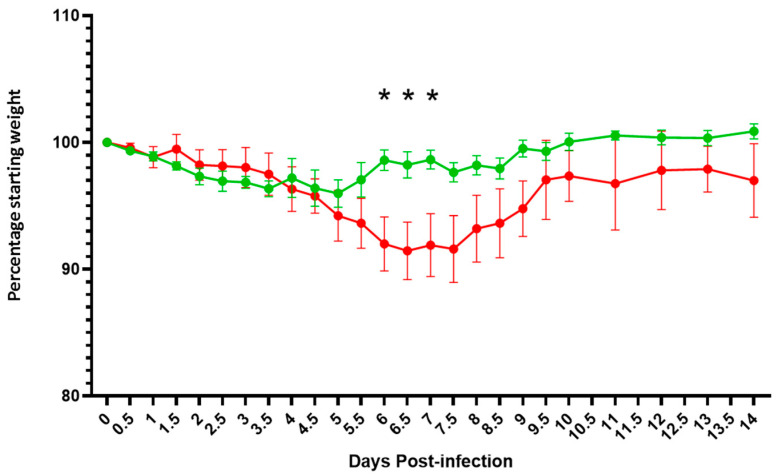
Average weight loss in both Syrian hamster groups, one immunized with VLP (green) and the other control (red), following live virus challenge. Animals were weighed twice a day (AM and PM) and the average weight and variation among the group plotted. Significant deviation between the groups (*p* < 0.05, ANOVA post-hoc Bonferroni) is indicated (*).

In keeping with the Syrian hamster model as published [50,51], both groups of animals lost weight for 5 days post virus challenge, but weight loss was arrested in the VLP group from day 6 whereas the control group continued to lose weight for a further 3 days. All animals recovered by 14 days post challenge but the difference in weight loss between the groups at 5–8 days post challenge was statistically significant (*p* < 0.05, ANOVA post-hoc Bonferroni) with the immunized animals recovering faster than the controls (Figure 9).

**Figure 10:**
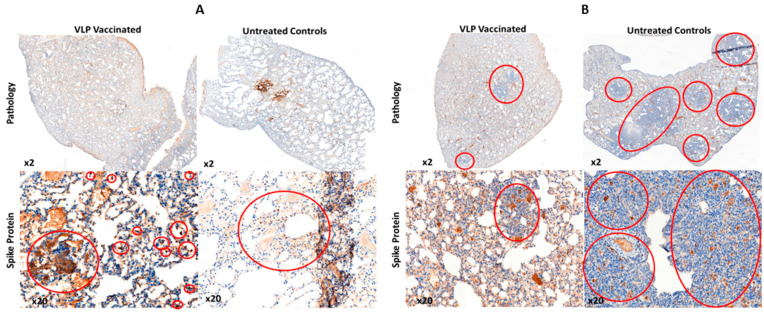
Histology of lung tissue taken from culled animal at 2 days (**A**) and 10 days (**B**) post live virus challenge. Upper panels—gross pathology revealed by H + E staining showing levels of lung sections showing vacuolization and eosinophilia (blue). Lower panels—immunohistochemistry with an anti-S antibody (brown).

Following post mortem, the lungs (right cranial and left) of selected animals in both groups were examined for overt signs of pathology, one animal each at 2 days post challenge, and two animals each at days 10 and 14 post challenge. At day 2 markers in the right cranial and left lung were similar for each animal although the single highest severity score, for alveolar wall necrosis, was found in the control animal. At day 10 the VLP treated group had much reduced overall scores, particularly for markers of inflammation, when compared to the control although the distinction between the groups was lost by day 14 (Table 1). Differential pathology was also apparent following histochemical staining the VLP group showing markedly reduced bronchiolar and alveolar inflammation compared with control animals at 2 days post challenge, a feature which was retained to the end of the monitoring period at 10 days post-challenge (Figure 10, upper panels). Immunohistochemistry for the presence of the S protein in areas of inflamed lung tissue showed clumps of S positive syncytial cells were present in both groups but that the overall level of S protein was much higher in the non-vaccinated animals when compared to those immunized with the VLPs at both of the time points tested (Figure 10, lower panels).

## 4. Discussion

The emergence of SARS-CoV-2 into the human population, most likely by spill over from a zoonotic reservoir [54], has led to the rapid development and licensing of several efficacious vaccines including those based on inactivated virus, adenovirus vectors, or direct nucleic acid [19,55,56]. Other experimental vaccines, including VLP and VLP-like vaccines have also been reported [48,57,58,59,60]. Based on a previously described VLP of SARS CoV-2 [9] we report here the development and test of a VLP for SARS-CoV-2 based on the co-expression of the S, M and E proteins using the baculovirus expression system. VLPs were shown to consist of the requisite CoV structural proteins, which were assembled into vesicle-like structures as shown by EM analysis. Further, these VLPs reacted with SARS-CoV-2 infected patient sera from two different serum sets, confirming their antigenicity and the combination of Western blot positivity, visualization and antigenic reactivity led to their use in trial immunizations. As an immunogen, non-adjuvanted SARS-CoV-2 VLPs generated a neutralizing antibody response in all immunized animals, which was boosted further following live virus challenge. No adverse events were recorded following immunization- consistent with previous tests of insect cells expressed material as vaccines [23,61]. Immunization with the SARS-CoV-2 VLPs did not prevent replication of the challenge virus but virus titers in the oral and nasopharyngeal samples were reduced when compared with the control group, the oral titers reaching statistical significance between the groups at 4 days post challenge. The SARS-CoV-2 VLPs used here were assembled using the Wuhan S protein but the challenge SARS-CoV-2 virus stock was the B1.1.7 S variant that carries the N501Y residue change [62], which has been reported to improve ACE-2 binding and partly evade the neutralizing antibody response [63,64]. In addition, the VLP was administered intradermally while the challenge virus was applied directly to mucosal surfaces in the nasal passage. Nevertheless, immunized animals demonstrated reduced weight loss and recovered from infection faster than control animals in a standard challenge model. At autopsy, substantially reduced pathology in the lungs of the immunized animals was apparent when compared with the controls with reduced eosinophilia and lower levels of virus encoded S protein in those few lesions present. These findings would be consistent with antibody levels being insufficient to prevent infection *per se* but sufficient to restrict virus proliferation and reduce the severity of disease overall. Similar data was obtained for the Ad26 based COVID-19 vaccine when tested in the Syrian hamster model [65]. Hennrich and co-workers have described a VLP vaccine for SARS-CoV-2 based on a mini-spike form of S delivered by a VSV based replicon which also prevented weight loss and overt disease in a similar model [49] and Tan et al. have described a novel thermostable RBD decorated VLP based on the SpyCatcher technology [57] which induced very high levels of neutralizing antibody consistent with protection although a formal virus challenge was not reported. Kang et al. also reported SpyCatcher enabled VLPs, with similar results [66]. Plescia et al. reported SARS-CoV-2 VLPs produced in mammalian cells by co-expression of S, M, N and E [12] while Xu et al. reported mammalian cell expressed VLPs with only S, M and E consistent with the data reported here [48]. Neither study investigated the immune response to the VLPs made. VLPs made by co-expression of all four structural proteins in mammalian cells, have been used to investigate the basis of genome packaging and, in turn, the role of variant sequences on the efficiency of SARS-CoV-2 virus assembly, providing evidence that amino acid changes in N rather than S are significant drivers of variant emergence [67]. Yilmaz et al. have also described VLPs from mammalian cells based on all four structural proteins and demonstrated superior immune responses following immunization of mice, rats and ferrets but these VLPs were adsorbed to alum and contained a CpG adjuvant prior to immunization [60]. The responses reduced but do not prevent challenge virus infection when used in a hACE2 transgenic (tg) mouse model but significantly reduced lung pathology, similar to the data reported here [60]. Mi et al. and Naskalska et al. reported VLPs made in insect cells by co-expression of S, M and E but investigated neither the antigenicity nor the immunogenicity of the materials described [43,59]. Insect cells expressed S1 protein was coupled to bacteriophage AP205 to produce a VLP-like structure by van Oosten et al. [58] that induced better immune responses in hACE2 tg mice than S protein alone although no challenge of the immunized mice was performed. Inactivated recombinant Newcastle Disease Virus (NDV) expressing a stabilized S protein, also a VLP-like structure, induced excellent immunity that protected against disease in the hamster model, with the virus load undetectable at 5 days post challenge [68], slightly faster than the kinetics of virus clearance reported here. However, the challenge inoculum in that study was lower than used here (10^4^ versus 1.5 × 10^5^ respectively). In our study, VLPs were immunogenic in the absence of adjuvant and were produced using a technology that is already in use for both human and animal vaccines offering scalability, an acceptable manufacturing process and an established route to licensure [23,58]. The VLPs described here have strong precedents in other examples derived from emerging enveloped viruses, including, among others, influenza [39,69], Ebola [70], HIV [71], Nipah [72] and SARS-CoV [9].

## Figures and Tables

**Table 1:** Pathology scores for hamster lungs. Lesions were scored for severity on a scale of 1–4 and colored accordingly.

	Day 2				Day 10								Day 14							
	S96 (VLP)		S101 (Ctrl)		S94 (VLP)		S95 (VLP)		S99 (Ctrl)		S100 (Ctrl)		S92 (VLP)		S93 (VLP)		S97 (Ctrl)		S98 (Ctrl)	
	Rcra	L	Rcra	L	Rcra	L	Rcra	L	Rcra	L	Rcra	L	Rcra	L	Rcra	L	Rcra	L	Rcra	L
Tissue affected (%)	85	90	40	90	15	85	80	70	85	65	100	80	70	85	95	95	100	75	55	30
Syncytial cells (Y/N)	Y(1)	N	N	N	Y(1)	Y(1)	Y(1)	N	Y(2)	Y(2)	Y(2)	Y(2)	N	N	N	N	Y(1)	Y(1)	Y(2)	Y(1)
I/A hemorrhage	0	0	0	1	0	2	0	0	3	2	1	1	1	0	0	0	0	0	0	0
I/A oedema	3	3	1	3	0	3	0	1	1	0	2	1	3	1	4	4	4	1	1	0
I/A fibrin	2	3	1	3	0	2	0	0	2	2	1	0	2	1	3	3	2	1	1	0
Hyaline MBs	0	0	0	1	0	0	0	0	0	0	0	0	0	0	1	1	1	0	0	0
Bronchiolar inflammation	0	0	0	0	0	2	2	0	3	4	3	3	0	0	0	0	0	2	2	1
Alveolar inflammation	2	2	1	0	2	3	2	1	2	4	4	3	1	1	0	0	1	3	2	2
Alveolar wall necrosis	0	3	1	4	1	2	1	0	1	1	2	1	0	0	4	3	3	1	0	0
Type II pneumocyte hyperplasia	0	0	0	0	0	1	0	0	1	4	4	3	0	0	0	0	0	3	1	2
Vascular necrosis	0	0	0	1	0	0	0	0	0	0	0	0	0	0	0	0	0	0	0	0
Thrombosis (Y/N)	N	N	N	N	N	N	N	N	N	N	N	N	N	N	N	N	N	N	N	N
Fibrosis	0	0	0	0	0	0	0	0	0	1	1	0	0	0	0	0	0	0	0	0
Hemosiderin	0	0	0	0	0	0	0	0	0	0	0	0	0	0	0	0	0	0	0	0

## Data Availability

Not applicable.
